# Narrowed host ranges do not constrain future host range expansion in RNA phage Φ6

**DOI:** 10.1128/jvi.00198-26

**Published:** 2026-05-27

**Authors:** Taylor P. Andrews, Abbey Isaac, Siobain Duffy

**Affiliations:** 1 Microbial Biology Graduate Program, Rutgers University 5970, New Brunswick, New Jersey, USA; 2 School of Environmental and Biological Sciences, Rutgers University 5970, New Brunswick, New Jersey, USA; 3 Department of Ecology, Evolution, and Natural Resources, School of Environmental and Biological Sciences, Rutgers University 5970, New Brunswick, New Jersey, USA; Michigan State University, East Lansing, Michigan, USA

**Keywords:** experimental evolution, host use, RNA virus, bacteriophage

## Abstract

RNA viruses frequently shift between different host species and emerge on novel hosts. Part of this evolutionary process can involve specialization, when viruses adapt to increase their fitness on a particular host, often at the expense of their ability to infect other hosts. This trajectory has the potential to lead to extreme host narrowing, which excludes all other previously accessible hosts. The consequences of the evolutionary history of host specialization on a virus’s future evolutionary potential are understudied. Using previously evolved specialized strains of model dsRNA bacteriophage Φ6, which had lost their ability to infect some hosts, we challenged specialists to adapt to their ancestral and other challenge hosts and identified resulting mutations. We found that these specialists readily re-gained their broader host ranges, at rates comparable to previously observed emergence events in Φ6, indicating a lack of potential constraint from their mutational backgrounds due to epistasis. While some viral strains achieved host range re-expansion by reversing the original host-narrowing mutations gained during specialization, others used secondary mutations, which were found to be parallel mutations previously associated with Φ6 entry into those host species. This study contributes to our understanding of the evolutionary dynamics of host shifting in RNA viruses and their strategies to re-expand following specialization, which is relevant to spillback events and recurring host shifts that are observed in nature.

**IMPORTANCE:**

The consequences of RNA virus specialization on a virus’s future evolutionary potential are not well understood. This study addresses the gap in the literature by investigating the mechanisms of viral host range re-expansion using a model RNA virus. We found that RNA viruses readily re-expand their host ranges to infect both novel and previously accessible hosts, both by mutational reversion and secondary mutations. Investigating these evolutionary mechanisms improves understanding of recurring host shifts and spillback events, which occur often in nature.

## INTRODUCTION

Over the course of their evolutionary histories, viruses frequently gain host range mutations and successfully adapt to infect new host species (1). These emergence events pose a growing threat to human health and agriculture (2). However, host shifts often impose a fitness cost on evolving viruses due to factors such as antagonistic pleiotropy. Research has shown that when a virus evolves to improve its fitness on a particular host, a common consequence is the reduction in its ability to infect other hosts (3–5). This can lead to a narrowing of host range, in which these emerging viruses lose the ability to infect some of their previous hosts altogether (6, 7).

These narrowed host ranges are unlikely to be permanent. While factors such as the accumulation of epistatic interactions over time may constrain the ability of viruses to reverse this trajectory (8), viruses can and do experience new host shifts. Indeed, both nature and laboratory experimentation have provided examples of viruses recurrently shifting between different hosts, including influenza (9) and coronaviruses (10). There is also a clear connection to the increasing number of studies that investigate viruses spilling back into previous hosts after having adapted to new hosts (SARS-CoV-2, flaviviruses, etc.) (11–13). However, these shifts involve fluctuating fitness on different hosts but not binary changes in host use; these studies do not examine viruses that reduced their host ranges to completely exclude previously accessible hosts.

By contrast, there is little research on how durable the narrowed host range of an emerging virus is. We have previously used the Φ6 model RNA virus system to examine how further host range mutation is constrained in viruses with expanded host ranges (14). We do not know whether specialization on a novel host constrains the future potential for a wider host range in RNA viruses. It is also unclear whether host range re-expansion is achieved by reversing the mutations that narrow host range, which can be adaptive on the host they are emerging on, or whether viruses need not experience “reverse evolution” (8, 15) and instead find new host range-expanding mutations at additional sites. One previous work by Crill et al. with the single-stranded DNA coliphage ΦX174 examined the effect of fluctuating between two hosts for moderate lengths of time and found that adaptation to one usually required reverse mutations to facilitate adaptation to the second (16). This seminal work was conducted with a small number of passages in an ssDNA virus. Similar studies have not been conducted in other systems; therefore, the generality of their results remains unknown. While many studies have explored the evolutionary effects of alternating host passaging, their passaging schemes are usually too brief to allow the viruses to specialize to one host before exposure to another (17–22), limiting their relevance to reverse evolution.

**Fig 1:**
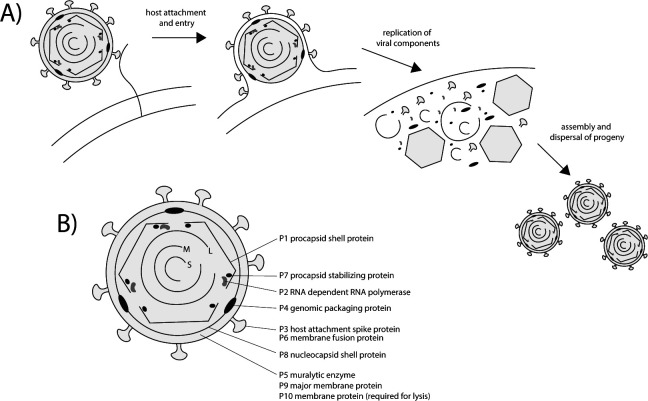
Φ6 life cycle and structure. (**A**) Major steps of the Φ6 life cycle. Following attachment to the host cell and fusion with the cell’s membrane, Φ6 enters the cell when the type IV pilus retracts and sends +ssRNA into the cytosol. Translation produces proteins that form offspring capsids. Single-stranded sense strands of the replicated genomes are packaged into the nascent capsids in a specific order (S, M, and finally L), leading to conformational changes to the capsid. As they enter the capsid, the genomic segments are complemented to become double stranded. Following completion of the assembly process and incorporation of a lipid membrane derived from the host’s inner membrane, viral progeny are released from the host via cell lysis. (**B**) Schematic of a Φ6 virion with select structural proteins identified. Proteins not depicted are P12 (morphogenic membrane protein) and P13 (minor membrane protein) (inspired by reference 23).

Φ6 is a well-studied model RNA virus. Previous research provides a detailed understanding of its life cycle, gene function, and host range adaptations (Fig. 1). Protein P3, the host attachment spike protein, has been shown to have a crucial role in host range determination and is very commonly observed to undergo mutation during host range adaptation (23).

In this study, we used the evolution of reduced host range (loss of plaque formation) during Φ6 adaptation to a distantly related novel host (6) to study the dynamics of how specialist viruses can broaden their host ranges. Three lineages, all sharing a common ancestor, were evolved for 30 passages (~150 generations; one passage equates to about 5 generations of viral replication [24, 25]) on a novel host, *Pseudomonas oleovorans* ERA, that is distantly related to the *Pseudomonas syringae* and *Pseudomonas savastanoi* strains its ancestors could infect. Three lineages found mutations that improved fitness on ERA, which simultaneously narrowed their host range. We forced emergence of these lineages on the two hosts they lost the ability to infect: *P. syringae* pv. *tomato* (three virus lineages), the standard laboratory host of Φ6, *P. savastanoi* pv. *phaseolicola* (one lineage, at both 20 days of passaging and 30 days of passaging on ERA). We similarly tested a novel host that the ancestor of the experiment could not infect, *P. syringae* pv. *atrofaciens* (three virus lineages). Our results show that RNA viral host range quickly re-expanded in these circumstances, both by reversion of host range-narrowing mutations and by additional, compensatory mutations.

## MATERIALS AND METHODS

### Strains and culture conditions

The previously described bacterial strains (26) were provided by Paul Turner (Yale University, New Haven, CT), who originally obtained *Pseudomonas savastanoi* pv. *phaseolicola* HB10Y (referred to hereafter as PP) from the American Type Culture Collection (no. 21781, Bethesda, MD), *P. syringae* pv. *tomato* (TOM) and *P. syringae* pv. *atrofaciens* (ATRO) from Gregory Martin (Cornell University, Ithaca, NY), and *P. oleovorans* ERA from Leonard Mindich (Public Health Research Institute, Newark, NJ). These isolates were all <90% genetically identical to each other (26). While it is not known whether all four hosts express a type IV pilus (the typical site of Φ6 attachment, Fig. 1), each possesses pilin-encoding genes. We identified the pilA proteins of these hosts by their similarity to experimentally verified *Pseudomonas* type IV pilus protein (GenBank L35847) (27) using NBCI tBLASTx (GenBank accession numbers XEN64223.1 [PP], XEO13889.1 [TOM], XEN74481.1 [ERA], and XEL06953.1 [ATRO]) and compared them using BLASTp (28).

Bacteria were grown overnight in LC medium (lysogeny broth, pH 7.5) at 25°C, 110 rpm. Phages were cultured with host bacteria using the pour plate technique, in 3 mL of 0.7% LC agar (top agar layer) on 1.5% LC agar plates, which were then incubated overnight at 25°C as previously described (29). High-titer lysates were obtained by scraping off the top agar layer (containing host bacterial lawn and >500 phage plaques), combining the top agar with 3 mL LC broth, and vortexing the mixture to liberate the phage before centrifugation for 10 minutes at 3,000 rpm. The supernatant was then passed through 0.22 μm filters to remove bacterial cells and yield high-titer phage stocks.

**Fig 2:**
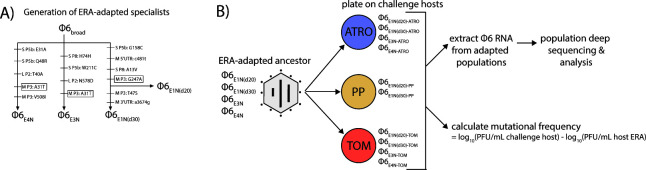
Generation of specialized ancestor populations and expanded host range mutants. (**A**) Generation of ERA-adapted specialist ancestors from reference 6. Specialist lineages subsequently used for host range re-expansion experiments are highlighted in yellow, and experimentally confirmed host range-narrowing mutations are boxed in black. (**B**) Experimental model for host range re-expansion of ERA-adapted specialists.

Four ERA-specialized strains [Φ6_E1N(d20)_, Φ6_E1N(d30)_, Φ6_E3N_, and Φ6_E4N_] were previously evolved, isolated, and sequenced (6). These strains infect host ERA and cannot form plaques on (i.e., detectably infect) hosts TOM (all four), ATRO (all four), or PP [Φ6_E1N(d20)_ and Φ6_E1N(d30)_] (Table 1) (6). The host range-narrowing mutation G247A is shared by common ancestry for Φ6_E1N(d20)_ and Φ6_E1N(d30)_ but A31T was found independently (parallel mutation) by Φ6_E3N_ and Φ6_E4N_. Narrowed host ranges of ERA-specialized strains were re-confirmed via triplicate spot plating 5 µL of serially diluted lysates onto lawns of ERA, TOM, ATRO, and PP. Plating high titers of these ERA-specialized strains on TOM, ATRO, and PP selects for mutants with a broader host range, simulating emergence on these hosts. The experimental design is illustrated in Fig. 2.

**TABLE 1:** Ancestor strain host ranges and host range-narrowing mutation

φ6 strain	Host range-narrowing mutation (in P3)	Host range of ancestor strain			
		ERA	PP	TOM	ATRO
φ6_broad_	None	•^*a*^	•	•	
φ6_E1N(d20)_	G247A	•			
φ6_E1N(d30)_	G247A	•			
φ6_E3N_	A31T	•	•		
φ6_E4N_	A31T	•	•		

^*a*^ A dot indicates that the phage can successfully plaque on the given host.

### Mutation frequency assays

For each of the four ERA-specialized strains, three replicate high-titer lysates were plated via pour-plate technique and titered on hosts ERA, ATRO, and TOM. Φ6_E1N(d20)_ and Φ6_E1N(d30)_ were also titered on host PP. The plaque-forming units (PFU) per milliliter of the lysates on challenge hosts were standardized against PFU per milliliter on host ERA (14):


$$Mutation\;frequency=log_{10}(PFU/mL{\;}_{challenge\;host})-log_{10}(PFU/mL{\;}_{host\;ERA})$$

Statistical analyses (analysis of variance and paired two-tailed Student’s *t*-test, where appropriate) were performed to compare mutation frequency of strains on the different challenge hosts. Analyses were conducted in Microsoft Excel (Redmond, WA) and visualized in R (version 4.2.2 [2022-10-31], http://www.r-project.org).

### Host range assay of reverted phages

The host ranges of Φ6 strains cultivated on challenge hosts TOM [φ6_E1N(d20)-TOM_, φ6_E1N(d30)-TOM_, φ6_E3N-TOM_, and φ6_E4N-TOM_], ATRO [φ6_E1N(d20)-ATRO_, φ6_E1N(d30)-ATRO_, φ6_E3N-ATRO_, and φ6_E4N-ATRO_], and PP [φ6_E1N(d20)-PP_ and φ6_E1N(d30)-PP_] were assessed via spot plating. High-titer lysates were serially diluted, and 5 μL volumes were spotted onto an LC top agar overlay inoculated with host bacteria (either ERA, TOM, ATRO, or PP). Evidence of plaquing at a dilution of 10^−6^ indicated an ability to successfully infect the host.

### Library preparation

Viral RNA was extracted from high-titer lysates of the ERA-adapted ancestor strains [φ6_E1N(d20)-ERA_, φ6_E1N(d30)-ERA_, φ6_E3N-ERA_, and φ6_E4N-ERA_] and strains grown on challenge hosts TOM [φ6_E1N(d20)-TOM_, φ6_E1N(d30)-TOM_, φ6_E3N-TOM_, and φ6_E4N-TOM_], ATRO [φ6_E1N(d20)-ATRO_, φ6_E1N(d30)-ATRO_, φ6_E3N-ATRO_, and φ6_E4N-ATRO_], and PP [φ6_E1N(d20)-PP_ and φ6_E1N(d30)-PP_] using QiaAMP Viral RNA MiniKit (Qiagen, Valencia CA). The extracted RNA was purified using 0.85% low-melting point agarose gel electrophoresis and Zymoclean Gel RNA recovery kit (Zymo Research, Irvine, CA) or Agarase (Thermo Fisher) according to the manufacturer’s directions. If necessary, RNA samples were ethanol precipitated to increase concentration (30).

RNA samples were sent to SeqCenter (Pittsburgh, PA) for cDNA reverse transcription synthesis (Maxima H Minus Double-Stranded cDNA Synthesis Kit) and Illumina sequencing (Illumina NovaSeq X Plus sequencer).

### Data analysis

Paired-end 2× 150 bp Illumina reads (SRA, PRJNA1172709) underwent demultiplexing, quality control, and adapter trimming using bcl-convert (v4.2.4, Illumina).

The reads were mapped to a WT-Φ6 reference genome (where all segments are concatenated into a single genome, https://zenodo.org/records/14516538) using BWA-MEM2 (Galaxy v2.2.1+galaxy0) with default settings (31, 32) (https://usegalaxy.org/). All viral samples had an average of between 275.5× and 87,901× read coverage to the Φ6 genome, determined using Samtools idxstats (Galaxy v2.0.5) (33).

### Identifying variants

VarScan (34) was used to detect single-nucleotide variants (SNVs) between consensus sequences of ERA-specialized ancestors and mutant populations successfully able to infect challenge hosts. BAM files from BWA-MEM2 (mapped to WT-Φ6 reference genome) were converted (Samtools mpileup [Galaxy v2.1.7]) (33), and VarScan was run to identify variants (compared to the WT-Φ6 reference genome) and estimate their frequencies (default settings, except minimum frequency to call homozygote = 0.01) (Galaxy v2.4.2) (34). The WT-Φ6 comparison VarScan results of ERA-adapted ancestors and challenge host strains were then compared by subtracting variant frequencies to identify sites of likely evolutionary selection within the genome. Sites with less than 100× coverage for either strain were omitted.

Aside from mutations in protein-coding regions of the Φ6 genome, some sites at the ends of the M and L segment had a ≥20% change in frequency (between 22% and 60%). The ends of the segments, especially the L segment, are sequenced at a much lower depth than the more central parts of the segments, which leads to misleadingly large changes in frequency due to small samples. We eliminated SNVs that had <100 reads either before or after exposure to the challenge host from further consideration in this text, but complete SNV details are given in File S2.

To identify sites with notable changes in polymorphism, the change in Shannon entropy within the Φ6 genome before and after exposure to a challenge host was calculated (14). Shannon entropy shows the levels of polymorphism at each site within the genome by analyzing absolute base coverage determined from deep sequencing, which complements the VarScan analysis using a consensus ancestral sequence.

To obtain genome nucleotide counts by position, BAM files (obtained as described above) were analyzed using Integrative Genomics Viewer IGVTools (igvtools count --windowSize 1 --bases) (35). Shannon entropy (*H*) was calculated for each position of the genome:


$$H\left(x\right)=\;-\sum_{i=1}^{n=4}P\left(x_{i}\right)\log_{2}P\left(x_{i}\right)$$

where *n* represents the four nucleotides and *P(x_i_*) represents the proportion of a single nucleotide over all those read at a certain position. The change in Shannon entropy between the ERA-adapted “ancestors” (*X_b_*) and challenge host mutant populations (*X_a_*) shows the change in polymorphic base composition at each site:


$$\Delta H=H\left(X_{a}\right)-H\left(X_{b}\right)$$

Sites with notably high |Δ*H*| were investigated manually using nucleotide count data to identify mutations of interest. The Shannon entropy changes at genome positions with less than 100× coverage for either sample were not considered.

Single-nucleotide variants identified through VarScan or change in Shannon entropy were then translated to determine the associated amino acid change (functional annotation of variants, using https://lab.siobain.com/fav/, in comparison to the wild-type concatenated Φ6 genome sequence, https://zenodo.org/records/14516538). Analyses were conducted and visualized in R (v4.2.2 [2022-10-31], http://www.r-project.org) unless otherwise noted.

### Broth infection assay

We also assessed whether the ERA-adapted ancestor strains could infect host TOM in liquid culture. Although our spot-plate host range assays did not show evidence of plaquing on soft agar, phage liquid culturing is an alternative method to demonstrate infectious ability (36). We attempted to observe replication on host TOM by coculturing a known number of phages (titered on permissive host ERA) with host TOM, and then titering on ERA to look for evidence of replication. Phage strains were combined with TOM host cells in liquid culture and assessed for evidence of replication. TOM cells added were in exponential phase; overnight liquid cultures were subcultured 1:100 and incubated for 5–6 hours at 25°C at 110 rpm. A half milliliter of this exponentially growing bacterial culture was mixed with 1,000 PFU of each phage in 0.5 mL of LC and incubated overnight at 25°C at 110 rpm. Cocultures were filtered using 0.22 μm filters to remove TOM host cells. The resulting viral extracts were titered via plaque assay on permissive host ERA to look for evidence of replication on host TOM (increased titer compared to the beginning of the experiment). Assays were done with six replicates, and titers were compared with paired one-tailed Student’s *t*-tests (alternative = “greater”) in R (v4.2.2 [2022-10-31], http://www.r-project.org).

## RESULTS

### Mutational frequency

**Fig 3:**
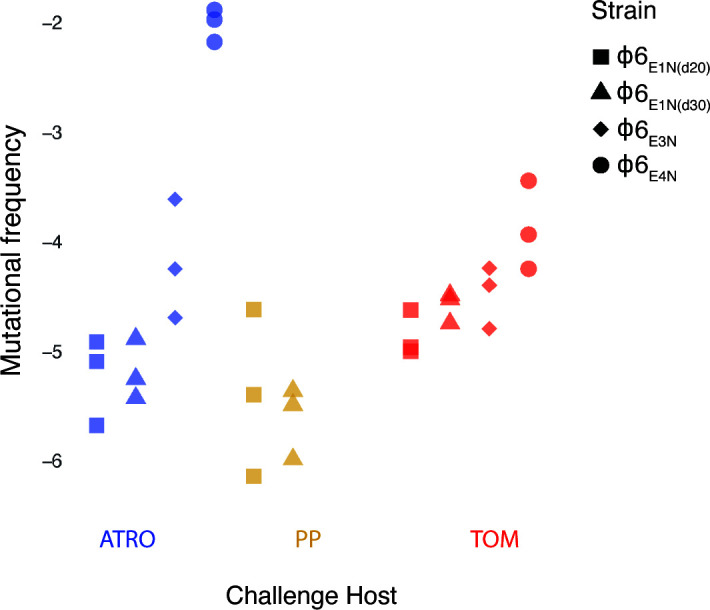
Mutational frequencies. Mutational frequency of ERA-adapted strains on challenge hosts ATRO (blue), PP (gold), and TOM (red). Results normalized against PFU per milliliter of same lysates on ERA (*n* = 3). Mutation frequency = log_10_(PFU/mL on challenge host) − log_10_(PFU/mL on ERA).

Serially diluted, titered lysates of ERA-adapted ancestor strains were plated on challenge hosts ATRO, PP, and TOM (PP was excluded from φ6_E3N_ and φ6_E4N_ challenge plating as they could already infect PP; see Table 1). With the exception of Φ6_E4N_ (*P* = 0.01, two-tailed Student’s *t*-test), each viral strain had similar mutational frequencies across the different challenge hosts (Fig. 3, mutational frequencies).

### Host range assay of reverted phages

The adapted viral populations were found to have (re)gained the ability to infect multiple hosts, not just the single host they were grown on (Table 2). The mutations necessary to infect host ATRO appeared to be the most specific.

**TABLE 2:** Host range assay of reverted phages^*a*^

φ6 strain		Host range			
		ERA	PP	TOM	ATRO
φ6_E1N(d20)_	TOM	•^*b*^	•	•	
	ATRO	•			•
	PP	•	•	•	
φ6_E1N(d30)_	TOM	•	•	•	
	ATRO	•			•
	PP	•	•	•	
φ6_E3N_	TOM	•	•	•	•
	ATRO	•	•	•	•
φ6_E4N_	TOM	•	•	•	•
	ATRO	•	•	•	•

^*a*^ The host ranges of adapted populations, determined via spot plating, are shown. Challenged φ6 populations are described according to their ancestral lineage (e.g., φ6_E3N_) and the host they were challenged on (TOM, ATRO, or PP).

^*b*^ A dot indicates that the phage can successfully plaque on the given host.

### Identification of host range re-expansion mutations

High-titer lysates of ERA-adapted ancestor strains (~1 × 10^10^ PFU/mL) were plated on challenge hosts, yielding host range mutant populations (1 × 10^5^ to 1 × 10^7^ PFU/mL). Both ancestral and post-challenge host lysates underwent population-wide deep sequencing, which allows for an effective “snapshot” of the mutational neighborhood (14). ERA-adapted ancestor and challenge host mutant populations were compared using VarScan to identify SNVs associated with host range expansion (File S2). Identified SNVs with a greater than 20% change in frequency between ancestral and mutant populations are shown in Table 3; all of these high-frequency non-synonymous SNVs were in the host attachment spike protein P3. Reversion of the original G247A host range-narrowing mutation in the φ6_E1N_ lineage was observed most of the time [in φ6_E1N(d20)-PP_, φ6_E1N(d20)-TOM_, and φ6_E1N(d30)-PP_]. Reversion of the A31T host range-narrowing mutation (present in the φ6_E3N_ and φ6_E4N_ lineages) occurred only in one population (Φ6_E3N-TOM_).

**TABLE 3:** High-frequency, non-synonymous SNVs with estimated variant-allele frequency above 20%^*a*^

φ6 strain	Challenge host	nt mutation	AA mutation	Change in frequency (%)
φ6_E1N(d20)_	ATRO	c4822t	A133V	98.95
	PP	c5164g	A247G*	95.01
	TOM	a4813g	Q130R	32.51
		c5164g	A247G*	23.00
φ6_E1N(d30)_	ATRO	c4822t	A133V	98.64
	PP	c5164g	A247G*	84.38
	TOM	a4813g	Q130R	58.53
φ6_E3N_	ATRO	c4822t	A133V	91.86
	TOM	a4515g	T31A*	37.80
φ6_E4N_	ATRO	c4822t	A133V	95.25
	TOM	a6085g	D554G	51.05

^*a*^ Nucleotide (nt) and translated amino acid (AA) mutations are noted, along with their change in frequency (single-nucleotide variant [SNV] frequency in adapted populations vs ancestral populations, detected by population-deep sequencing). Reversions are indicated with asterisks (*).

Outright reversion of the original host range-narrowing mutations of these ERA-adapted lineages was not always observed; other mutational pathways in the P3 protein occurred. For all four lineages, P3 mutation A133V was identified as the only coding SNV in mutants able to grow on the novel host ATRO. For the φ6_E1N_ lineages, the Q130R mutation appeared to be important for re-expansion into host TOM. In lieu of a reversion of the original A31T host range-narrowing mutation (observed in Φ6_E3N-TOM_), Φ6_E4N_ was observed to re-expand into host TOM through mutation D554G.

### Identification of sites with high levels of polymorphism

**Fig 4:**
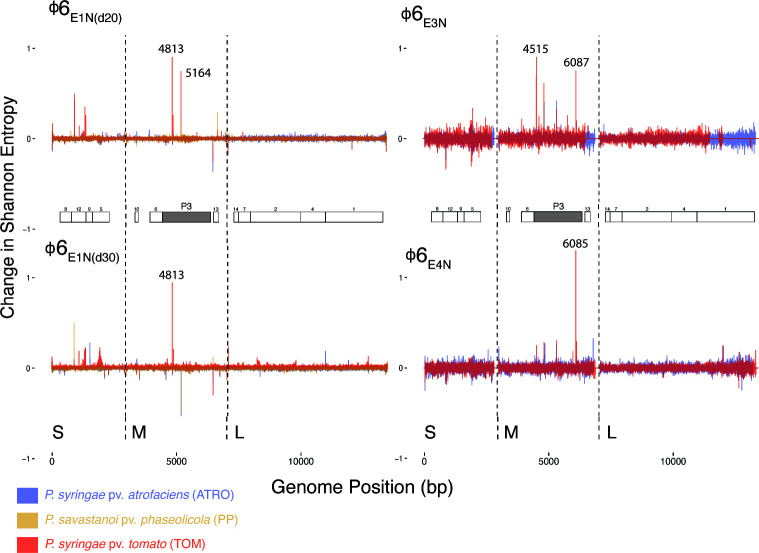
Change in Shannon entropy following host range expansion. Each strain’s graph shows the overlapping change in Shannon entropy (Δ*H*) data sets on challenge hosts ATRO (blue), PP (gold), and TOM (red). Data were aligned to a concatenated Φ6 genome, with different genomic segments (S, small; M, medium; L, large) delineated using dashed lines. Protein-coding regions are also shown with the P3 spike protein-coding region highlighted in gray. Genomic sites of interest (with high change in Shannon entropy) are annotated next to the associated peaks with the site number in the concatenated genome.

The change in Shannon entropy (representing levels of polymorphism) of each site within the genome was calculated and compared between ancestor and mutant populations to determine sites that changed in their degree of polymorphism following re-expansion of the host range (Fig. 4 ;File S1).

Across all the challenge host strains, sites with the largest changes in polymorphism following host range expansion were located within the P3 protein-coding region.

The sites with peaks in Δ*H* generally corresponded with the SNVs identified in the VarScan analysis (Table 3), with the exception of φ6_E3N-TOM_, which showed a peak at site 6087, which is still within the P3 protein-coding region. This site appears to have undergone some diversification, where 100% of the ERA-adapted ancestor population had a cytosine at this site, but the TOM population had detectable polymorphism (4.6% a, 84.7% c, and 10.7% t). These site mutations translate to L555I (c6087a) and L555F (c6087t), the latter of which has been previously associated with expansion into host ERA (4).

Consistent with the results in Table 3, Fig. 4 also highlights the fact that SNVs were found in lower frequencies in the TOM populations compared to the other challenge hosts. The SNVs that affected host range expansion on PP and ATRO were present in over 90% of the mutant populations, whereas TOM-adaptive SNVs were found in only 37%–58% in the TOM-adapted populations (Table 3).

While all of the high-frequency SNVs were found in the gene for P3, only 28% of the SNVs in protein-coding genes above a 1% frequency were found in P3. Other genes that contained high numbers of SNVs included lytic protein P5a (12% of coding SNVs), morphogenic membrane protein P12 (11% of coding SNVs), and the procapsid shell protein P1, which contained a notable number of SNVs for the φ6_E3N_ and φ6_E4N_ lineages (26% and 18%).

### Broth infection assay

Because our results on TOM showed less decisive shifts to a single non-synonymous substitution than on hosts ATRO and PP, we investigated whether TOM was already being infected by our strains but just failing to form plaques. If so, host range mutations would not be as essential for forming plaques on TOM compared to these other hosts. Assays (*n* = 6) did not show statistically significant increases in phage titers on TOM in broth culture, indicating that the barrier to infection on TOM is more significant than just whether or not plaques can form on agar plates (*P* values ≥0.24 for three strains, but *P* = 0.081 for φ6 _E3N_).

Instead, another factor for less decisive SNV results on TOM is that there are more mutational pathways to re-enter TOM, including multiple SNVs that were below our original 20% frequency threshold for SNV analysis. We looked at all non-synonymous SNVs at above 1% frequency on strains grown on TOM (Table 4), many of which arose in parallel in different lineages. These SNVs help account for the lower overall frequencies of TOM-adaptive SNV frequencies initially discussed, increasing the frequencies of viruses with any non-synonymous mutation in P3 in TOM-grown populations to 58%–87%.

**TABLE 4:** TOM-adapted non-synonymous single-nucleotide variants^*a*^

φ6 strain	nt mutation	AA mutation	Protein-coding region	Change in frequency (%)
φ6_E1N(d20)_	a1328g	K192R	P12	5.90
	a4813g	Q130R	P3	32.51
	c5164g	A247G*	P3	23.00
φ6_E1N(d30)_	a4813g	Q130R	P3	58.53
φ6_E3N_	a4515g	T31A*	P3	37.80
	a4813g	Q130R	P3	12.50
	c5320g	S299W	P3	5.76
	a6085g	D554G	P3	11.46
	c6087t	L555F	P3	9.28
φ6_E4N_	a4813g	Q130R	P3	5.04
	c5320g	S299W	P3	5.70
	a6085g	D554G	P3	51.05
	c6087t	L555F	P3	12.19

^*a*^ Reversions are indicated with asterisks (*).

### Differences in host attachment target

We compared candidate pilA proteins from our four hosts (Table 5). This comparison demonstrates the genetic variation (which may confer morphological differences) between the putative receptor proteins on these bacterial hosts; such differences could contribute to the range of adaptive mutations we observed in this study.

**TABLE 5:** Percent identity comparison of *Pseudomonas* host pilin protein sequences

Pilin	PP (%)	TOM (%)	ERA (%)	ATRO
PP	–			
TOM	76.32	–		
ERA	59.09	47.30	–	
ATRO	42.62	45.58	54.69	–

## DISCUSSION

This study demonstrates that the model dsRNA virus Φ6 can re-expand its host range following evolved specialization and loss of host range through both reverse evolution and additional mutations. Mutational frequency experiments demonstrated the ease with which these specialists could revert to re-expand their host ranges, at similar rates to other host range evolution studies (14). This may reflect that the specialized strains only had one mutation which conferred the host narrowing phenotypes. The original experiment which yielded these specialists spanned a “moderate” timescale (30 days, about 150 generations), which may not have been long enough for epistatic constraints to emerge (6, 8). RNA viruses can have substantial epistatic interactions, such as between compensatory mutations and drug resistance mutations (37, 38), but these host range mutations have a far smaller effect on fitness than many of the antiviral resistance alleles.

We also explored the mutational mechanisms by which Φ6 achieved host range re-expansion. All coding SNVs included in the main analysis (>20% frequency, Table 3) were located in the P3 host attachment spike protein, which has been previously associated with host range expansion in Φ6 (4, 6, 14, 23, 25, 29, 39). The importance of spike proteins as the main—or sole—determinant of viral host range has been widely observed because of their role in host attachment (40–42). Similarly, the ability of single mutations to cause host range broadening in a wide range of viruses has been experimentally demonstrated (43). Other genomic regions that contained notable numbers of SNVs (above 1% frequency) included P5a, P12, and P1. The P5a lytic protein may contribute to host range in Φ6 because of its role in both phage entry and exit from the host cell. Mutations in proteins P5a and P12 have been previously associated with host range expansion (14), likely through their influence on phage exit from the cell, similar to the influence of neuraminidase on influenza host range (44). P1, the procapsid major structural protein (45), has not previously been associated with host range expansion in Φ6. It is possible that the SNVs identified on P1 are the result of genetic hitchhiking (46).

We observed multiple instances of mutational reversion, consistent with previous studies on viral adaptation to different hosts (16), including that the two isolates from a single lineage (φ6_E1N_), could not re-expand host range back to the typical laboratory host, PP, without reversion of the G247A site in P3. This reversion is caused by a transversion mutation, which is rarer than transitions due to mutational bias (4, 47), underscoring its importance in PP host infection. For all other hosts, we observed at least one compensatory mutation that achieved host range re-expansion without reversion of the original host narrowing mutations. Many of these compensatory mutations have been identified to confer fitness on the relevant hosts in previous Φ6 experimental evolution studies (14, 25, 29). The A133V mutation was one of only two possible host range mutations that allow ATRO infection in the ancestor of all of these Φ6 lineages, φ6_broad_ (14, 29). The Q130R mutation has been associated with adaptation to TOM in past work (25), along with the known host range mutation D554G (29). Such repeated observation of the same parallel mutations occurring during emergence on different host species is common in viral experimental evolution (48, 49). Furthermore, the prevalence of some of these mutations in the Illumina sequencing data indicates they were essentially the only pathway found in our study for re-infection of hosts ATRO and PP (14).

By contrast, there were not the same hard selective sweeps in the populations able to re-infect TOM. The highest frequency substitutions did not rise to >60% frequency in any of the four lineages (Table 3), which suggested there are more mutations that allow the strains to re-enter TOM compared to the other hosts tested. These other variants would have been present at <20% frequency but would be visible in Fig. 4. For φ6_E4N_, this included D554G and for φ6_E3N_ L555F, sites that have previously been implicated in expanded host range of Φ6 onto host ERA (4). However, the frequency of even two host range-expanding alleles in the populations growing on TOM does not add up to a comparably high frequency as in the populations re-emerged on ATRO and PP. Because the populations on TOM were behaving differently, we investigated whether these ERA-adapted strains were able to infect TOM at some low level at the start of the experiment but were just not able to form plaques, our method of detecting infection. Further experiments did not show that the host range-narrowed genotypes were able to infect TOM in broth, though future work with more sensitive techniques, such as qPCR, may be able to detect low levels of infection (50). Regardless, plaquing is a reasonable threshold for determining host range. It has a long, established history in phage biology (36, 51, 52) and mimics the multiple rounds of infection in a multicellular eukaryotic host needed to cause infection (53, 54).

The mechanism for entry of Φ6 into its usual laboratory host, PP, has been well studied. Phage protein P3 binds quite reversibly to the pilA of the type IV pilus (39). The pilus retracts, bringing the virion in contact with the outer membrane of the host bacterium, and the membranes fuse, liberating the lysis protein, which creates a small hole in the host cell wall such that the nucleocapsid can pass through and ultimately enter the cytosol (55). It is assumed that a similar mechanism of entry exists for Φ6 on related *Pseudomonas* strains, but the mechanisms of entry have not been demonstrated, and related cystoviruses can attach to rough LPS instead (56, 57). The limit to coinfection is more than 10 times higher on ERA than PP (58), suggesting other means of entry, but others in the literature assume that ERA must express a type IV pilus (4). Our observation of a limited range of mutational pathways utilized to enter ATRO (Table 3) could be connected to the increased genetic distance of its pilin protein in comparison to other hosts (Table 5), or the presence of other host range mutations present already in the ancestral P3 (14).

The mutations which conferred the ability to (re)infect the host the viral population was challenged on were observed to also extend to other bacterial hosts (Table 2). This could support the assumption of similar entry mechanisms into different hosts discussed above but could also be a result of other secondary mutations; for example, low frequencies of the P3 S299W mutation, a known ATRO-adaptive mutation (14), were observed in φ6_E3N-TOM_ and φ6_E4N-TOM_. It has been established that mechanisms of exit, not just entry, are often required for effective plaquing on ATRO (14). Our results highlight the need for further research on the mechanism of how this bacterium is infected by phi6.

Previous studies that looked to contrast the levels of mutational reversion and compensatory mutations did not examine host specialization; typically, they focused on overall fitness improvement, often after the introduction of deleterious mutations (59–61). We find that host specialization follows the same trends as these previous works; while reversion of some host-narrowing mutations may be necessary, most of the time, additional substitutions are sufficient. This does not mean that epistasis plays a limited role in host re-expansion. The limited range of host range-broadening mutations indicates that far fewer sites were available to these specialist lineages than the wild-type ancestor, similar to our previous work on serial host shifting in Φ6 (14). Consistent with their known capacity for rapid evolution and frequent host shifts (1), specialized RNA viruses can quickly broaden their host ranges by either reverse mutation or additional substitutions.

## Data Availability

All nucleotide sequencing data are available at NCBI under BioProject no. PRJNA1172709.

## SUPPLEMENTAL MATERIAL

File S1 jvi.00198-26-s0001.xlsb Nucleotide counts from all sequencing, with analysis for Shannon Entropy.

File S2 jvi.00198-26-s0002.xlsx VarScan outputs, with calculations used in manuscript.

ASM does not own the copyrights to Supplemental Material that may be linked to, or accessed through, an article. The authors have granted ASM a non-exclusive, world-wide license to publish the Supplemental Material files. Please contact the corresponding author directly for reuse.
